# Supplementary material for: A conserved differentiation programme facilitates inhibitory neuron production in the developing mouse and human cerebellum
Source: Development. 2025 Dec 19;152(24):dev204811. doi: 10.1242/dev.204811 (PMC12766576; doi:10.1242/dev.204811)
Supplement: Supplementary information [file develop-152-204811-s1.pdf]

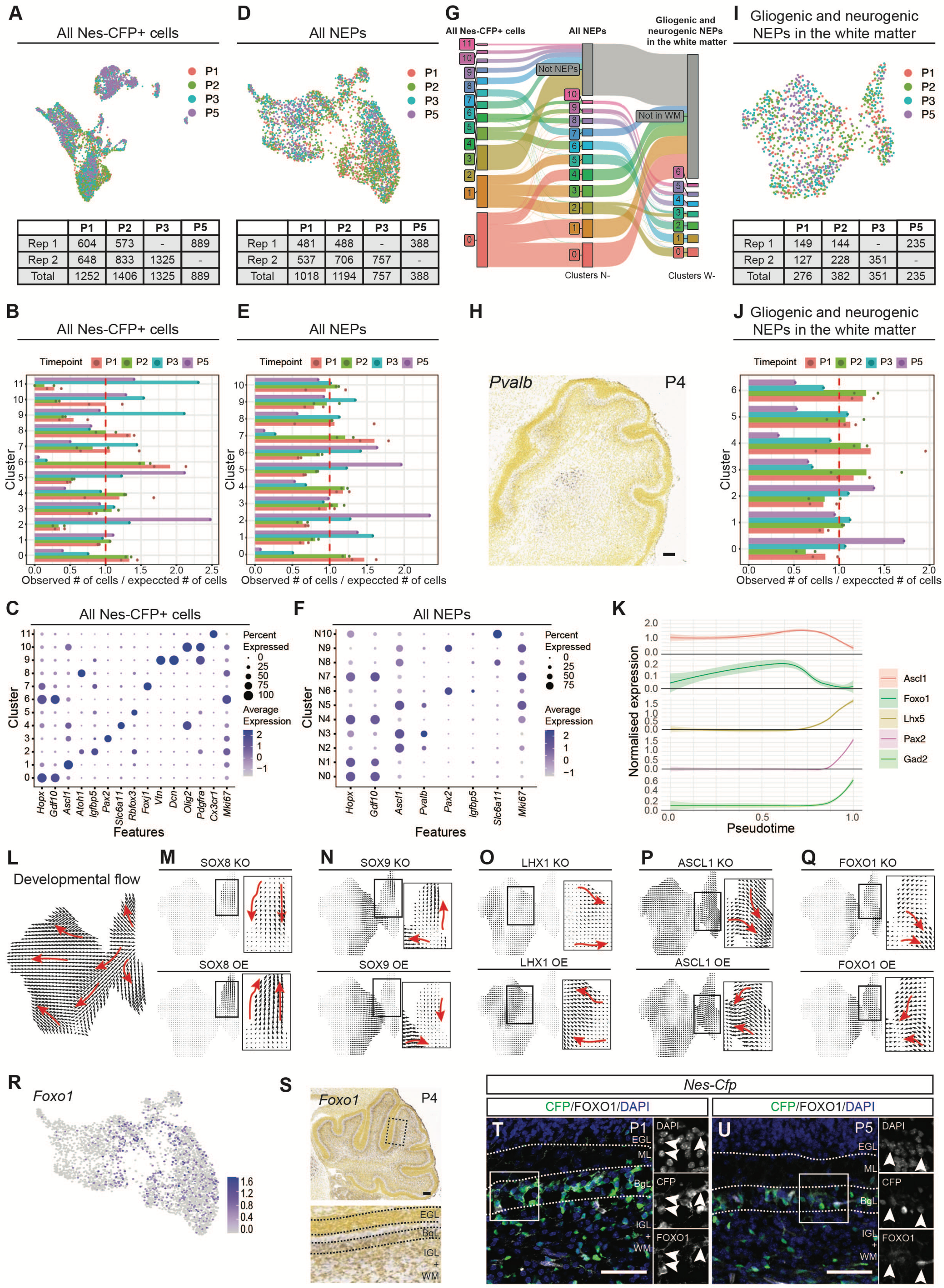

Fig. S1. Analyses of scRNA-seq of NEPs identify *Foxo1* as a possible regulator of NEP-to-inhibitory neuron differentiation *in vivo*.

(A) Uniform Manifold Approximation and Projection (UMAP) visualisation of all Nes-CFP^{+} cells coloured by sample timepoint and a table with the number of cells from each replicate at each timepoint. **(B)** Distribution of cells from different timepoints

within clusters from Nes-CFP+ cells. The y-axis shows the observed number of cells divided by the expected number of cells in each cluster. The dashed line indicates a ratio of 1. **(C)** Dotplot of established lineage markers used to annotate clusters in the Nes-CFP+ subset. The colour gradient signifies the normalised expression. The size of the circle indicates the percentage of cells expressing the specific marker within a cluster. **(D-F)** Similar to (A-C) but performed on the subset containing all NEPs. **(G)** Sankey plot showing the relationship between clusters across subsets. **(H)** Allen Brain Atlas P4 RNA *in situ* hybridisation of *Pvalb*. **(I-J)** Similar to (A-B) but performed on the subset containing only the gliogenic and neurogenic NEPs in the white matter. **(K)** Normalised expression of genes with respect to pseudotime. The shaded area signifies the 95% confidence interval. **(L)** Cellular trajectories computed by CellOracle using the non-perturbed data. The root cell was chosen from cluster W4. **(M-Q**) CellOracle simulation of either *in silico* knockout or overexpression of SOX8 (M), SOX9 (N), LHX1 (O), ASCL1 (P) and FOXO1 (Q). The effect on subpopulations is shown in the enlarged insets. Red arrows are added for visual guidance. **(R)** UMAP of all NEPs coloured by normalised expression of *Foxo1*. **(S)** Allen Brain Atlas P4 RNA *in situ* hybridisation of *Foxo1*. High magnification inset of the expression in the lobules is shown in the bottom panel. **(T-U)** Immunofluorescent analysis of CFP and FOXO1 on P1 (T) and P5 (U) *Nes-Cfp*/+ mice. Arrowheads indicate CFP+ cells with cytoplasmic FOXO1 expression. EGL: external granule layer, IGL: internal granule layer, ML: molecular layer, BgL: Bergmann glia layer, WM: white matter. Scale bars: 100 µM, except for T-U (50 µM).

Development • Supplementary information

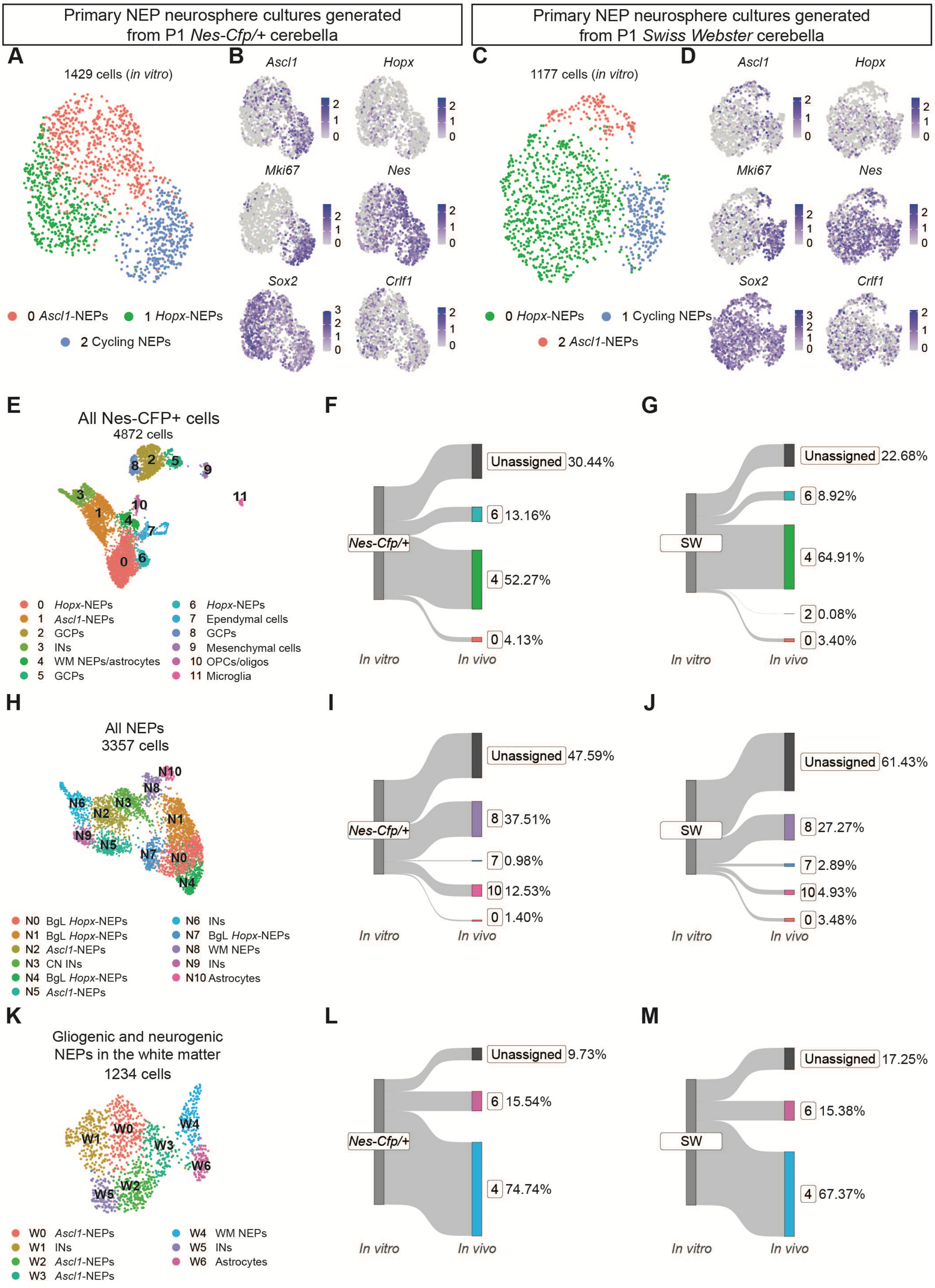
Primary NEP neurosphere cultures generated from P1 Nes-Cfp/+ cerebella
Primary NEP neurosphere cultures generated from P1 Swiss Webster cerebella
A
1429 cells (in vitro)
0 Ascl1-NEPs
1 Hopx-NEPs
2 Cycling NEPs
B
Ascl1
Hopx
Mki67
Nes
Sox2
Crlf1
C
1177 cells (in vitro)
0 Hopx-NEPs
1 Cycling NEPs
2 Ascl1-NEPs
D
E
All Nes-CFP+ cells
4872 cells
0 Hopx-NEPs
1 Ascl1-NEPs
2 GCPs
3 INs
4 WM NEPs/astrocytes
5 GCPs
6 Hopx-NEPs
7 Ependymal cells
8 GCPs
9 Mesenchymal cells
10 OPCs/oligos
11 Microglia
F
Nes-Cfp/+
Unassigned 30.44%
6 13.16%
4 52.27%
0 4.13%
In vitro
In vivo
G
SW
Unassigned 22.68%
6 8.92%
4 64.91%
2 0.08%
0 3.40%
H
All NEPs
3357 cells
N0 BgL Hopx-NEPs
N1 BgL Hopx-NEPs
N2 Ascl1-NEPs
N3 CN INs
N4 BgL Hopx-NEPs
N5 Ascl1-NEPs
N6 INs
N7 BgL Hopx-NEPs
N8 WM NEPs
N9 INs
N10 Astrocytes
I
Unassigned 47.59%
8 37.51%
7 0.98%
10 12.53%
0 1.40%
J
Unassigned 61.43%
8 27.27%
7 2.89%
10 4.93%
0 3.48%
K
Gliogenic and neurogenic NEPs in the white matter
1234 cells
W0 Ascl1-NEPs
W1 INs
W2 Ascl1-NEPs
W3 Ascl1-NEPs
W4 WM NEPs
W5 INs
W6 Astrocytes
L
Unassigned 9.73%
6 15.54%
4 74.74%
M
Unassigned 17.25%
6 15.38%
4 67.37%

Fig. S2. scRNA-seq of two independent primary NEP neurosphere cultures generated from P1 mouse cerebella and mapping of this dataset to the scRNA-seq of Nes-CFP$^+$ cells freshly isolated from neonatal mouse cerebellum and to the subsequent subclusters (all NEPs and WM NEPs).

(A-D) UMAP of 1429 and 1177 cells from two independent primary NEP cultures established from a P1 *Nes-Cfp/+* (A-B) or *Swiss Webster* (C-D) mouse, respectively. The data highlights the cellular diversity within the primary mouse NEP neurosphere cultures and the similarities between the two independent primary cultures. 3 clusters were detected in both, representing NEP subtypes and the proliferative cells. UMAPs of normalised expression levels of known marker genes further highlight the expression of *Ascl1* and *Hopx*, along with endogenous expression of *Nes* and *Sox2*, as pan-NEP markers (B and D). *Atoh1* and *Barhl1* expression were not observed, confirming little to no granule cell progenitor contamination under these culture conditions. **(E-M)** The two primary NEP neurosphere culture datasets (A and C) were mapped onto the *in vivo* scRNA-seq data of all Nes-CFP+ cells isolated from the neonatal cerebellum (E-G), and the subsequent iterative subclustering of all NEPs (H-J) and gliogenic and neurogenic NEPs in the white matter (K-M) to identify transcriptional similarities. **(F-G)** Sankey plots showing the mapping of the primary *Nes-Cfp/+* (F) and *Swiss Webster* (G) NEP neurosphere cultures to the *in vivo* all Nes-CFP$^+$ cells data (E). **(I-J)** Sankey plots showing the mapping of the primary *Nes-Cfp/+* (I) and *Swiss Webster* (J) NEP neurospheres cultures to all NEPs subset of the *in vivo* dataset (H). **(L-M)** Sankey plots showing the mapping of the primary *Nes-Cfp/+* (L) and *Swiss Webster* (M) NEP neurosphere cultures to the subset containing gliogenic and neurogenic NEPs in the WM (K). Cells with a prediction score lower than 0.6 were designated as "Unassigned".

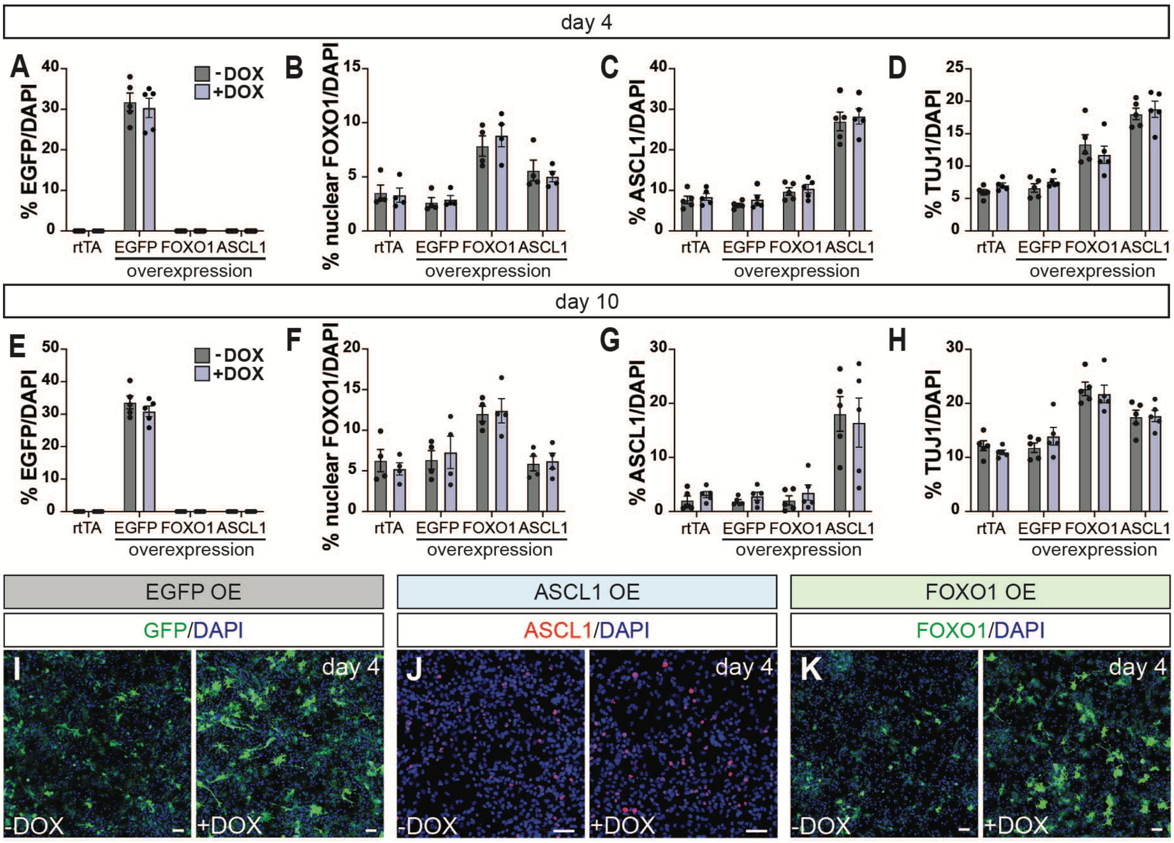

Fig. S3. The tetracycline-inducible promoter is leaky in primary NEPs.

(A-H) Quantification of EGFP$^+$, nuclear FOXO1$^+$, ASCL1$^+$ and TUJ1$^+$ cells at day 4 (A-D) and day 10 (E-H) of differentiation in rtTA NEPs or rtTA NEPs overexpressing either EGFP, FOXO1 or ASCL1, with and without doxycycline (DOX) treatment. (Multiple Wilcoxon tests, n=5 (except for B and F, n = 4), adjusting for multiple comparisons using the two-stage step-up method of Benjamini, Kreiger and Yukutieli). **(I-K)** Immunofluorescent analysis of NEPs overexpressing EGFP (EGFP OE) (I), ASCL1 (ASCL1 OE) (J) or FOXO1 (FOXO1 OE) (K) on day 4 of differentiation. Representative images are shown. Graphs show mean ± s.e.m. Scale bars: 50 µm.

Development • Supplementary information

A

Condition	Day	Total peaks	Minimum width	Maximum width	Width mean	Width median
Dam-alone	2	6023	333	1391	530.77	484
ASCL1-Dam	2	11267	291	1509	430.84	378
FOXO1-Dam	2	9171	249	1739	361.53	313
Dam-alone	6	3449	271	1789	440.90	391
ASCL1-Dam	6	2537	292	1354	453.64	396
FOXO1-Dam	6	4211	310	1941	487.97	422

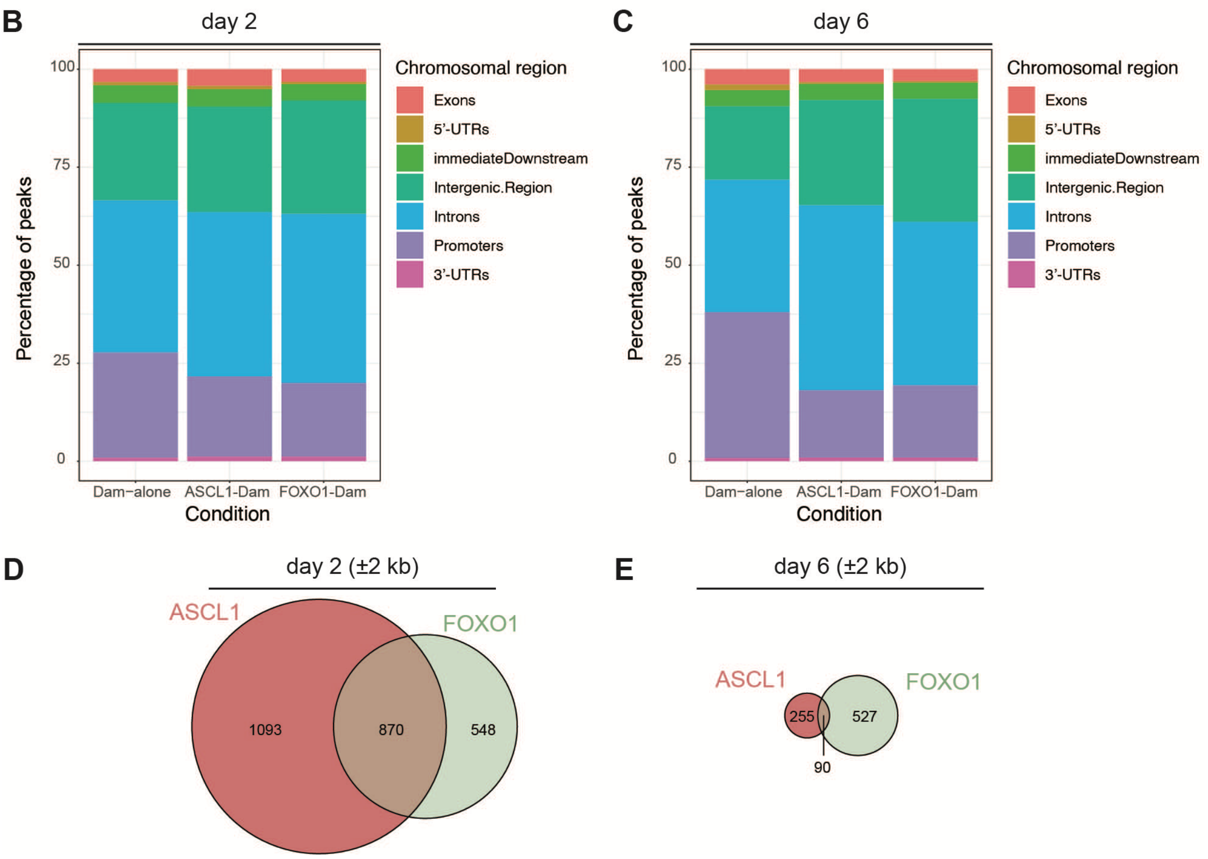

Fig. S4. Characterisation of the distribution and properties of peaks from ASCL1-Dam, FOXO1-Dam and Dam-alone during *in vitro* NEP differentiation. **(A)** Summary statistics of peak properties of ASCL1-Dam, FOXO1-Dam and Dam-alone at day 2 and day 6 of *in vitro* differentiation. **(B-C)** Classification of the chromosomal regions containing ASCL1-Dam, FOXO1-Dam and Dam-alone peaks at day 2 (B) and day 6 (C) of *in vitro* differentiation. **(D-E)** Venn diagrams of genes associated with significant and reproducible peaks of ASCL1-Dam and FOXO1-Dam on day 2 (D) and 6 (E) of differentiation (Table S4). Genes with peaks ±2kb from their transcriptional start site were used for the analysis. An overlap of 34.65% and 10.32% was observed at day 2 and day 6, respectively. For all analyses, significant peaks occurring in a minimum of ¾ replicates were used.

Development • Supplementary information

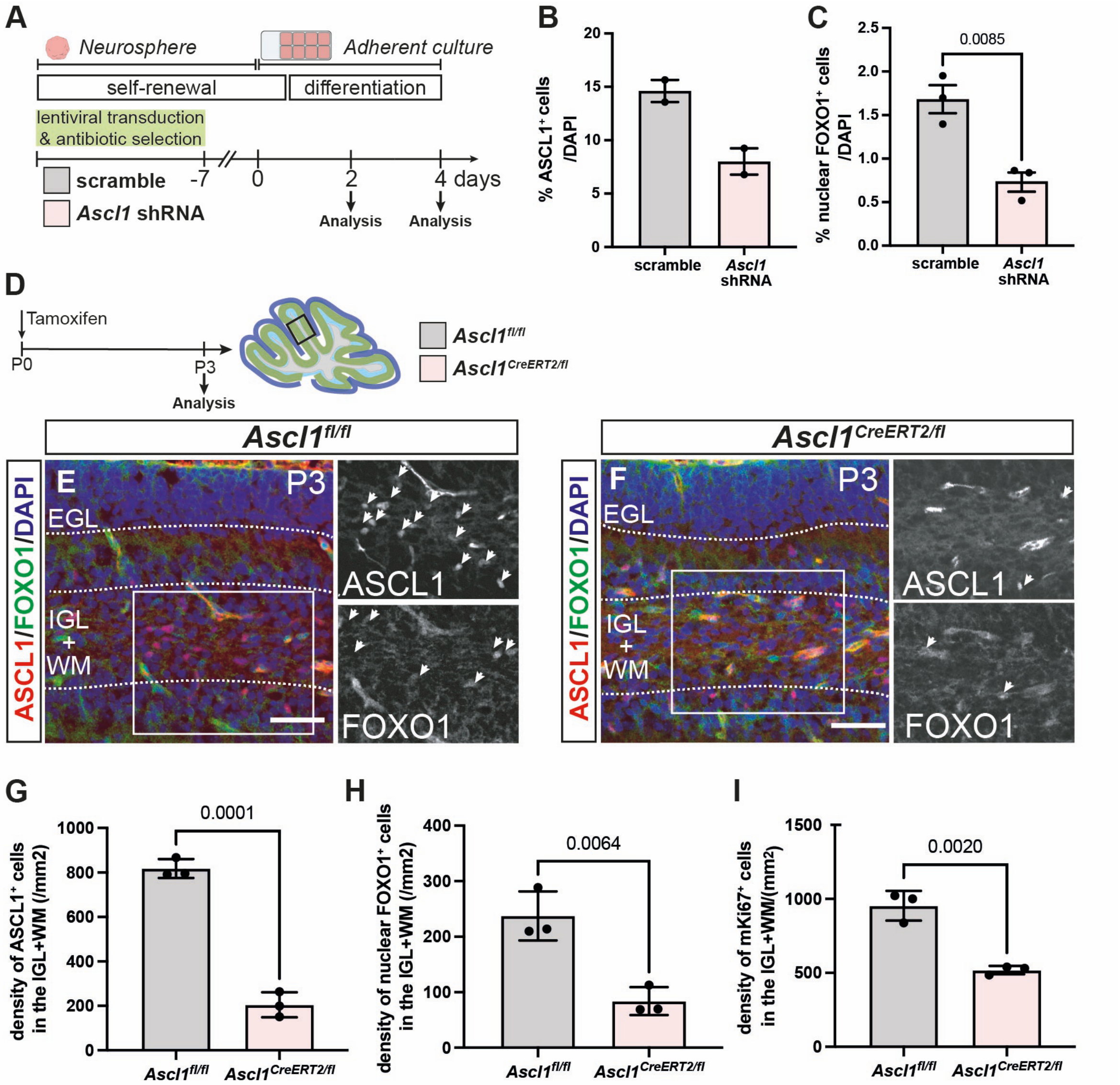

Fig. S5. *In vitro* and *in vivo* *Ascl1* loss-of-function leads to reduced FOXO1 levels. (A) Experimental plan. **(B)** Quantification of the percentage of ASCL1^{+} cells following shRNA-mediated knockdown (n=2) at day 2 of *in vitro* differentiation. Day 2 was analysed to capture peak ASCL1 levels during differentiation. **(C)** Quantification of the percentage of nuclear FOXO1^{+} cells following ASCL1 knockdown at day 4 of differentiation shows a significant reduction in nuclear FOXO1^{+} cells compared to the scramble control shRNA (n=3, paired t-test, p=0.0085). Day 4 was analysed to capture peak FOXO1 levels during differentiation. **(D)** Experimental setup. *Ascl1$^{CreERT2/fl}$* animals were given tamoxifen at P0, and the cerebella were analysed at P3 for ASCL1, FOXO1 and mKi67 levels. *Ascl1$^{fl/fl}$* animals were used as controls. **(E-F)** Immunofluorescent analysis of conditional knockout animals and their littermate controls (n=3 brains/condition). **(G-I)** Quantification of the density of ASCL1 (G), nuclear FOXO1 (H) and mKI67 (I) positive cells in the IGL and WM of conditional knockout and control cerebella (n=3 brains, unpaired t-test, G: p<0.001, H: p=0.0064, I: p<0.0020). Lobule 3 was analysed. Arrows show ASCL1^{+} or nuclear FOXO1^{+} cells. B-C: Graphs show mean ± s.e.m. G-I: Graphs show mean ± s.d. Scale bar: 50 µm

Development • Supplementary information

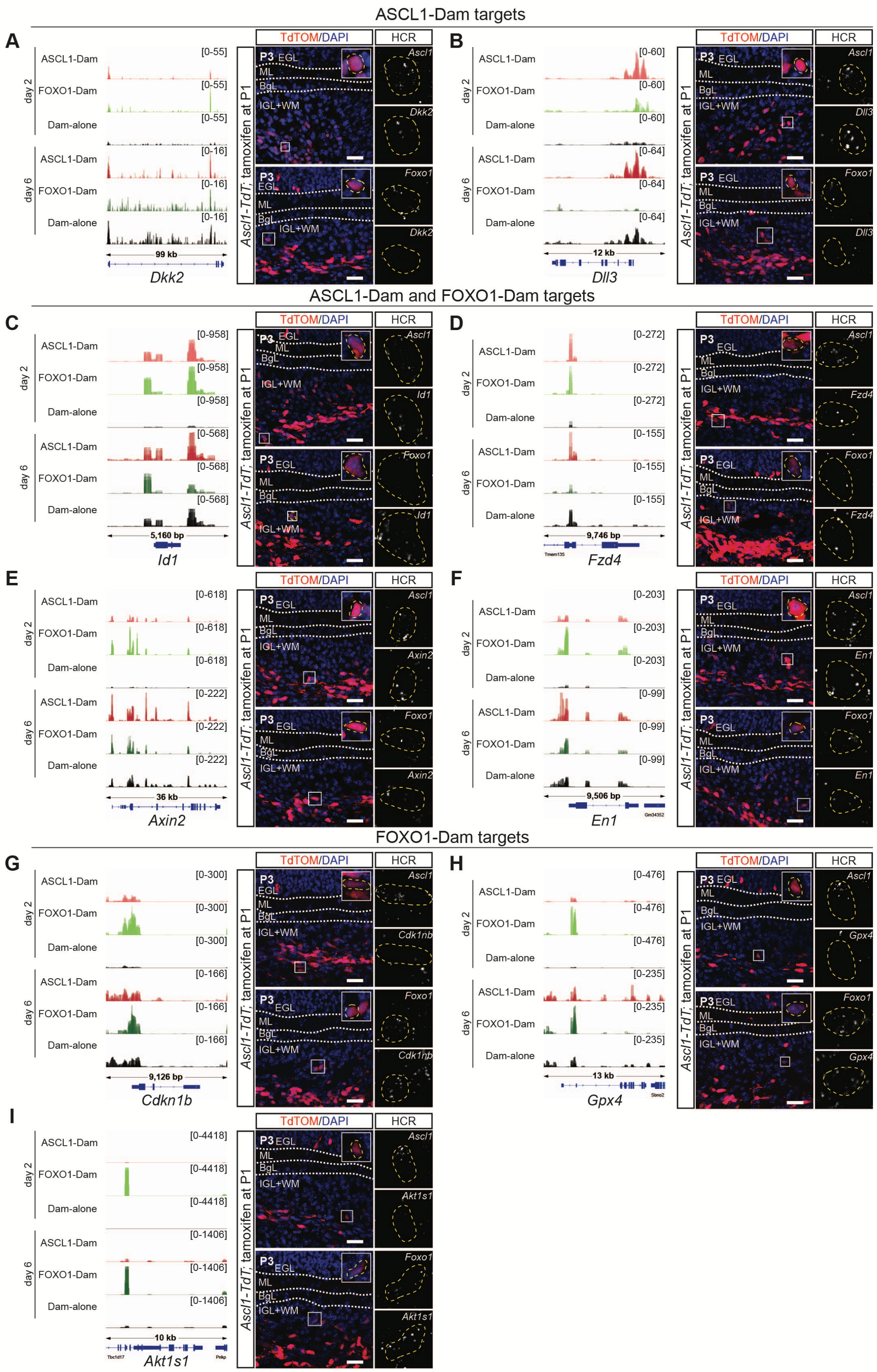

Development • Supplementary information

Fig. S6. *In situ* HCR analysis shows coexpression of ASCL1 and/or FOXO1 targets with the respective transcription factors within the *Ascl1*-NEP lineage.

(A-I) ASCL1-Dam, FOXO1-Dam and Dam-alone tracks at day 2 and 6 of *in vitro* differentiation are shown for ASCL1-Dam target gene loci (*Dll3* (A) and *Dkk2* (B)), ASCL1-Dam and FOXO1-Dam shared target gene loci (*Id1* (C), *Fzd4* (D), *Axin2* (E) and *En1* (F)) and FOXO1-Dam target gene loci (*Cdkn1b* (G), *Gpx4* (H) and *Akt1s1* (I)). Each track is an overlay of the 4 replicates. *In situ* HCR analysis on P3 *Ascl1-TdT* brains that were given Tamoxifen at P1 allows identification of *Ascl1*-NEP progeny via the native TdTOM expression. Multicolour *in situ* HCR for *Ascl1* (top) and *Foxo1* (bottom), together with each target gene, highlights the expression of ASCL1-Dam targets with only *Ascl1* (A-B), FOXO1-Dam targets with only *Foxo1* (G-I) and shared targets with both (C-F). Individual TdTOM^{+} cells are shown in high magnification insets, and the yellow dashed outline represents the outline of the cell analysed. Representative images are shown. Scale bars: 25 μM.

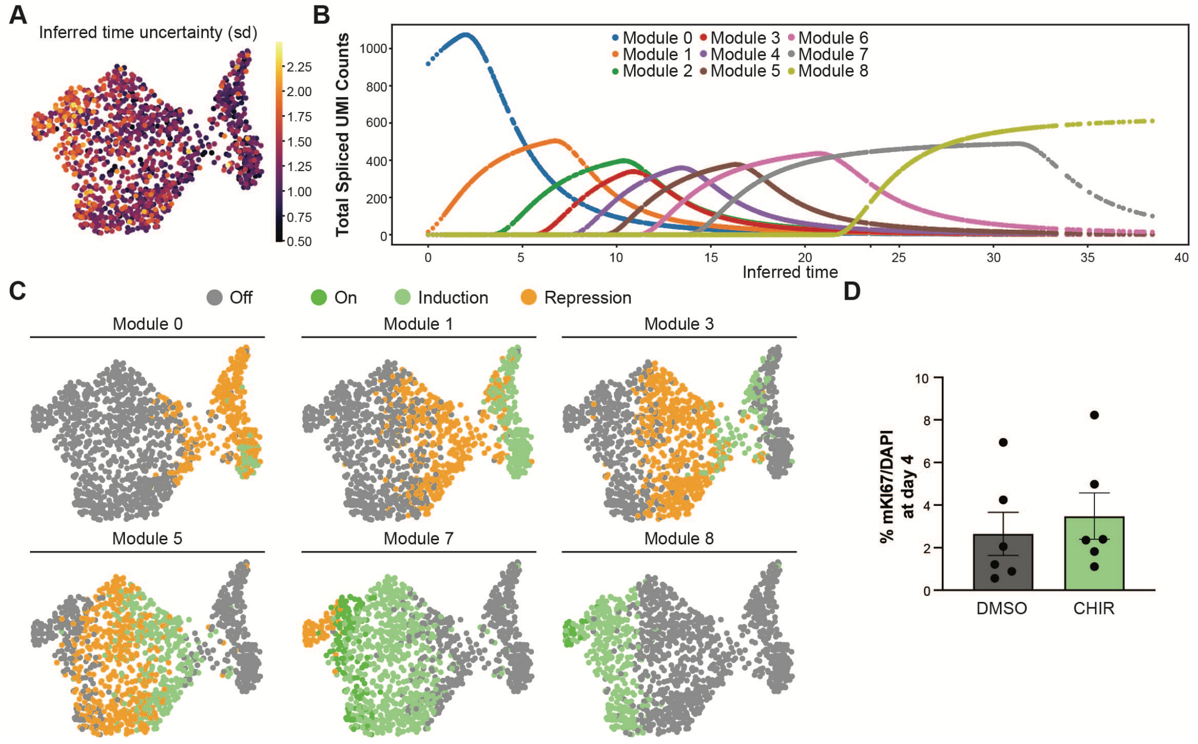

Fig. S7. Cell2Fate captures sequentially activated modules during NEP-to-inhibitory neuron differentiation.

(A) UMAP of WM NEPs labelled by inferred time uncertainty computed by Cell2Fate. **(B)** Total spliced UMI counts from each Cell2Fate module with respect to the computed inferred time. (**C)** UMAPs of WM NEPs labelled by the module state (Off, On, Induction, Repression) of modules 0, 1, 3, 5, 7 and 8. **(D)** Quantification of $mKI67^{+}$ cells at day 4 of differentiation in cultures treated with either DMSO or CHIR (ratio paired Student's t-test, n=6). Graphs show mean ± s.e.m.

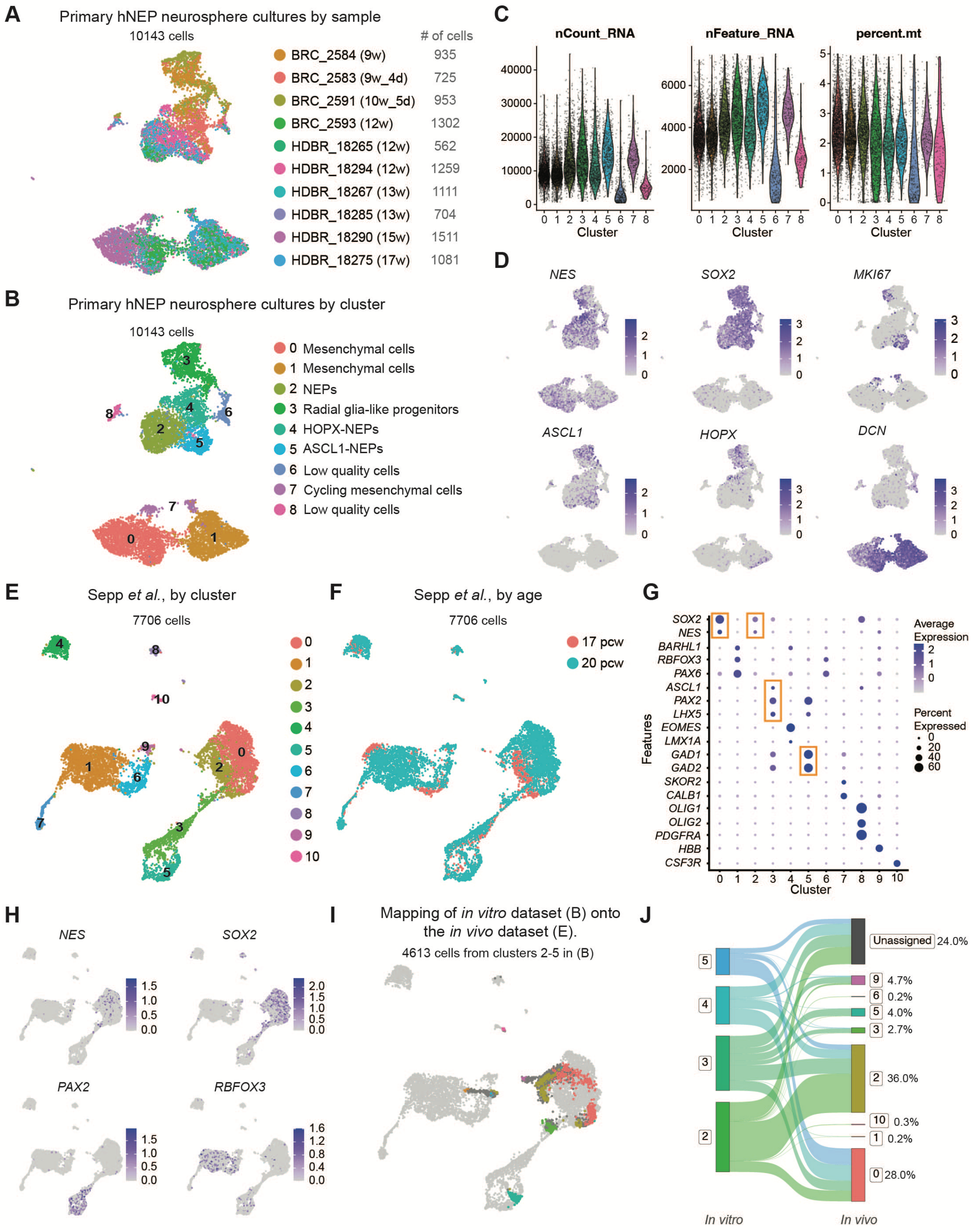

Fig. S8. scRNA-seq of primary hNEP neurosphere cultures generated from fetal human cerebella shows cellular diversity and mapping to human fetal cerebellum data (Sepp *et al*., 2024) highlights the similarities between hNEP neurosphere cultures and their *in vivo* counterparts.

(A-B) UMAP of 10143 primary hNEP neurosphere cultures labelled by donor (A) or cluster (B). Primary hNEP cultures were established from 10 individual donors aged 9-17 post-conception weeks. **(C)** Violin plots of cell quality metrics: nCount_RNA (total UMIs in a cell), nFeature_RNA (number of unique features expressed in a cell) and percent.mt (percent mitochondrial reads in a cell). Clusters 6 and 8 were removed from

downstream analysis due to their low quality. **(D)** UMAP of normalised gene expression of known marker genes. *NES, SOX2, ASCL1* and *HOPX* mark ventricular zone-derived cerebellar progenitors, including hNEPs, *MKI67* proliferating cells and *DCN* mesenchymal cells. Clusters 2-5, which show endogenous *NES*, *SOX2* and/or *ASCL1* and *HOPX* expression, were used for downstream analysis. (**E-F)** A subset of the Sepp *et al*. (2024) snRNA-seq data set was utilised for downstream analysis. A UMAP of 7706 cells from 17 and 20 pcw human fetal cerebella labelled by cluster (E) or post-conceptional age of the donor (F) shows the presence of expected cerebellar cell types at those stages. **(G)** Dotplot of established lineage markers used to annotate the Sepp *et al.* clusters. The colour gradient signifies the normalised expression. The size of the circle indicates the percentage of cells expressing the specific marker within a cluster. Lineage markers for clusters 0, 2, 3 and 5 are highlighted by an orange box. Based on marker gene expression, these clusters likely represent hNEPs (cluster 0 and 2, *NES*$^{+}$/*SOX2*$^{+}$/*ASCL1*$^{+}$), immature (cluster 3, *ASCL1*$^{+}$/*PAX2*$^{+}$/*LHX5*$^{+}$) and mature inhibitory neurons (cluster 5, *GAD1/2* $^{+}$). **(H)** UMAP of normalised expression of marker genes, dividing the Sepp *et al.* data into 3 major populations. *NES*$^{+}$/*SOX2*$^{+}$ ventricular zone-derived progenitors (clusters 0 and 2), *PAX2*$^{+}$ immature interneurons (clusters 3 and 5) and *RBFOX3*$^{+}$/ *PAX6*$^{+}$ rhombic lip-derived excitatory neurons (clusters 1 and 6). **(I)** Clusters 2-5 from the *in vitro* primary hNEP neurosphere data (B) were mapped to the Sepp *et al.* data (E) using the 200 top variable genes. UMAP of Sepp *et al*. data in grey with mapped *in vitro* cells coloured according to the colours of the most similar *in vivo* cluster. Cells with a prediction score lower than 0.6 were designated as “Unassigned”. **(J)** Sankey plot showing the relationship between *in vitro* and *in vivo* clusters, highlighting that the majority of the primary hNEP neurosphere cultures resemble *NES/SOX2* expressing cells *(in vivo* clusters 0 and 2, where ~65% of the *in vitro* data maps onto) and exhibit some inhibitory neuron characteristics (*in vivo* cluster 5).

Development • Supplementary information

Table S1. The number of cells in each condition, the marker genes used for cluster annotations and the lists of differentially expressed genes in each cluster obtained from the iterative subclustering of scRNA-seq data of Nes-CFP$^+$ cells isolated from neonatal cerebella from Pakula *et al.,* 2025.

Available for download at
https://journals.biologists.com/dev/article-lookup/doi/10.1242/dev.204811#supplementary-data

Table S2. Regulon specificity score for individual pySCENIC iterations (n=10) and summary of top 3 regulons and the frequency that they were identified.

Available for download at
https://journals.biologists.com/dev/article-lookup/doi/10.1242/dev.204811#supplementary-data

Table S3. Differentially expressed genes in each cluster obtained from the scRNA-seq of two primary mouse NEP neurosphere cultures.

Available for download at
https://journals.biologists.com/dev/article-lookup/doi/10.1242/dev.204811#supplementary-data

Table S4. Lists of motifs enriched in targeted DamID data identified by HOMER. For ASCL1-Dam and FOXO1-Dam conditions, HOMER was run on all significant peaks detected after normalisation to their Dam-alone control. For Dam-alone, HOMER was run on all significant peaks detected. For ASCL1-Dam and FOXO1-Dam co-regulated genes, HOMER was run on all reproducible Dam-alone peaks associated with genes, which also have reproducible and significant ASCL1-Dam and FOXO1-Dam ±1/2kb from their transcriptional start site. The analyses were done identically at day 2 and day 6.

Available for download at
https://journals.biologists.com/dev/article-lookup/doi/10.1242/dev.204811#supplementary-data

Table S5. List of genes with significant peaks identified by targeted DamID and GO term analysis of those genes.

Available for download at
https://journals.biologists.com/dev/article-lookup/doi/10.1242/dev.204811#supplementary-data

Table S6. Top 200 genes in each Cell2Fate module and GO term analysis using those genes.

Available for download at
https://journals.biologists.com/dev/article-lookup/doi/10.1242/dev.204811#supplementary-data

Table S7. Differentially expressed genes in clusters obtained from the scRNA-seq of primary hNEP neurosphere cultures and the reanalysis of the 17 and 20 pcw human fetal cerebella snRNA-seq from Sepp *et al.,* 2024.

Available for download at
https://journals.biologists.com/dev/article-lookup/doi/10.1242/dev.204811#supplementary-data

Table S8. List of primary and secondary antibodies used for immunofluorescent analyses.

**Primary antibodies**		
Antibody	Catalogue number, supplier	Dilution
Rabbit anti-FOXO1	#2880, Cell Signaling	1:200
Mouse anti-ASCL1	556604, BD Bioscience	1:500/1:333
Rabbit anti-TUJ1	ab18207-100ug, Abcam	1:1000
Chicken anti-GFP	ab13970, Abcam	1:2000
Goat anti-SOX9	#AF3075, R&D Systems	1:500
Goat anti-GAD1	#AF2086, R&D Systems	1:250
Mouse anti-GAD2	AB_2314499, DSHB	1:100
Mouse anti-PVALB	195004, Synaptic Systems	1:500
Rabbit anti-HOPX	HPA030180-100ul, Merck	1:1000
Mouse anti-MKI67	ab279653, Abcam	1:500
Goat anti-SOX2	AF2018, R&D Systems	1:1000
Chicken anti-GFAP	Ab4674-50ul, Abcam	1:1000
Goat anti-PAX2	#AF3364, R&D Systems	1:250
Goat anti-LHX5	#AF6290-SP, R&D Systems	1:200
**Secondary antibodies**		
Antibody	Catalogue number, supplier	Dilution
Donkey anti-rabbit Alexa Fluor™ Plus 488	#32790, ThermoFischer Scientific	1:500
Donkey anti-mouse Alexa Fluor™ Plus 555	#32773, ThermoFischer Scientific	1:500
Donkey anti-goat Alexa Fluor™ Plus 488	15930877, FisherScientific	1:500
Donkey anti-mouse Alexa Fluor™ Plus 647	15927745, FisherScientific	1:500
Donkey anti-chicken Alexa Fluor™ Plus 488	17777517, Fisher	1:500
Donkey anti-rabbit Alexa Fluor™ Plus 647	16239260, FisherScientific	1:500
Donkey anti-rabbit Alexa Fluor™ Plus 555	A32794, ThermoFisher	1:500
Donkey anti-mouse Alexa Fluor™ Plus 488	#32790, ThermoFischer Scientific	1:500
Streptavidin, Alexa Fluor™ 555 Conjugate	S32355, ThermoFisher	1:500
DAPI	MBD0015-1ML, Sigma-Aldrich	1:1000

Table S9. Summary of the statistical comparisons.

Figure	Test Performed	p-value	Multiple comparisons	
Figure 2F	Ordinary one-way ANOVA	$F_{(5, 46)}$ = 9.439 P<0.0001	Tukey's multiple comparisons	
			Comparisons	Adjusted p value
			0 vs 2	0.9909
			0 vs 4	0.8883
			0 vs 6	0.0726
			0 vs 10	<0.0001
			0 vs 14	0.0004
			2 vs 4	0.9966
			2 vs 6	0.2474
			2 vs 10	0.0004
			2 vs 14	0.0027
			4 vs 6	0.5131
			4 vs 10	0.0017
			4 vs 14	0.011
			6 vs 10	0.1287
			6 vs 14	0.4701
			10 vs 14	0.9526
Figure 2L	Ordinary one-way ANOVA	$F_{(5, 12)}$ = 7.9 P=0.0016	Tukey's multiple comparisons	
			Comparisons	Adjusted p value
			0 vs 2	>0.9999
			0 vs 4	0.9283
			0 vs 6	0.0028
			0 vs 10	0.0632
			0 vs 14	0.3845
			2 vs 4	0.9364
			2 vs 6	0.003
			2 vs 10	0.0663
			2 vs 14	0.3985
			4 vs 6	0.0133
			4 vs 10	0.2733
			4 vs 14	0.8789
			6 vs 10	0.4637
			6 vs 14	0.0817
			10 vs 14	0.8308
Figure 2R	Ordinary one-way ANOVA	$F_{(5, 46)}$ = 7.720 P<0.0001	Tukey's multiple comparisons	
			Comparisons	Adjusted p value
			0 vs 2	0.0689
			0 vs 4	0.9948
			0 vs 6	0.9986
			0 vs 10	0.2087
			0 vs 14	0.0938
			2 vs 4	0.0182
			2 vs 6	0.0257

Development • Supplementary information

<table>
<tr><td></td><td></td><td></td><td><table>
<tr><td>2 vs 10</td><td>0.0001</td></tr>
<tr><td>2 vs 14</td><td><0.0001</td></tr>
<tr><td>4 vs 6</td><td>>0.9999</td></tr>
<tr><td>4 vs 10</td><td>0.4615</td></tr>
<tr><td>4 vs 14</td><td>0.2678</td></tr>
<tr><td>6 vs 10</td><td>0.3891</td></tr>
<tr><td>6 vs 14</td><td>0.212</td></tr>
<tr><td>10 vs 14</td><td>>0.9999</td></tr>
</table></td></tr>
<tr><td>Figure 2S</td><td>Ordinary one-way ANOVA</td><td>$F_{(5, 46)}$ = 3.575
P=0.0082</td><td>Tukey’s multiple comparisons<table>
<tr><td>Comparisons</td><td>Adjusted p value</td></tr>
<tr><td>0 vs 2</td><td>>0.9999</td></tr>
<tr><td>0 vs 4</td><td>0.1229</td></tr>
<tr><td>0 vs 6</td><td>0.0286</td></tr>
<tr><td>0 vs 10</td><td>0.2105</td></tr>
<tr><td>0 vs 14</td><td>0.9106</td></tr>
<tr><td>2 vs 4</td><td>0.1843</td></tr>
<tr><td>2 vs 6</td><td>0.0474</td></tr>
<tr><td>2 vs 10</td><td>0.293</td></tr>
<tr><td>2 vs 14</td><td>0.9632</td></tr>
<tr><td>4 vs 6</td><td>0.9895</td></tr>
<tr><td>4 vs 10</td><td>>0.9999</td></tr>
<tr><td>4 vs 14</td><td>0.6247</td></tr>
<tr><td>6 vs 10</td><td>0.9837</td></tr>
<tr><td>6 vs 14</td><td>0.267</td></tr>
<tr><td>10 vs 14</td><td>0.749</td></tr>
</table></td></tr>
<tr><td>Figure 2T</td><td>Ordinary one-way ANOVA</td><td>$F_{(5, 46)}$ = 1.5
P=0.1918</td><td></td></tr>
<tr><td>Figure 3C</td><td>Row matched one-way ANOVA</td><td>$F_{(1,4)}$ = 149.0
p-value = 0.0003</td><td>Tukey’s multiple comparisons comparing the mean of every column.
EGFP OE vs FOXO1 OE: adjusted p-value = 0.0006
EGFP OE vs ASCL1 OE: adjusted p-value = 0.0006
FOXO1 OE vs ASCL1 OE: adjusted p-value = NA</td></tr>
<tr><td>Figure 3E</td><td>Row matched one-way ANOVA</td><td>$F_{(1.332,5.328)}$ = 138.6
p-value = <0.0001</td><td>Tukey’s multiple comparisons comparing the mean of every column.
EGFP OE vs FOXO1 OE: adjusted p-value = 0.0501
EGFP OE vs ASCL1 OE: adjusted p-value = 0.0005</td></tr>
</table>

Development • Supplementary information

			FOXO1 OE vs ASCL1 OE: adjusted p-value = 0.0006
Figure 3G	Row matched one-way ANOVA	$F_{(1.024,3.073)}$ = 35.83 p-value = 0.0087	Tukey's multiple comparisons comparing the mean of every column.<br>EGFP OE vs FOXO1 OE: adjusted p-value = 0.0172<br>EGFP OE vs ASCL1 OE: adjusted p-value = 0.0072<br>FOXO1 OE vs ASCL1 OE: adjusted p-value = 0.0278
Figure 3J	Row matched one-way ANOVA	$F_{(1.722,6.889)}$ = 39.25 p-value = 0.0002	Tukey's multiple comparisons comparing the mean of every column.<br>EGFP OE vs FOXO1 OE: adjusted p-value = 0.0319<br>EGFP OE vs ASCL1 OE: adjusted p-value = 0.0024<br>FOXO1 OE vs ASCL1 OE: adjusted p-value = 0.0184
Figure 3K	Row matched one-way ANOVA	$F_{(1.472,5.888)}$ = 17.37 p-value = 0.0044	Tukey's multiple comparisons comparing the mean of every column.<br>EGFP OE vs FOXO1 OE: adjusted p-value = 0.0017<br>EGFP OE vs ASCL1 OE: adjusted p-value = 0.1287<br>FOXO1 OE vs ASCL1 OE: adjusted p-value = 0.1207
Figure 3N	Ratio paired t-test	Scrambled vs shRNA1 : p-value = 0.0059<br>Scrambled vs shRNA2 : p-value = 0.0033	
Figure 3P	Ratio paired t-test	Scrambled vs shRNA1	

Development • Supplementary information

		: p-value = 0.0245<br>Scrambled vs shRNA2 : p-value = 0.0317	
Figure 3Q	Ratio paired t-test	Scrambled vs shRNA1 : p-value = 0.8809<br>Scrambled vs shRNA2 : p-value = 0.8511	
Figure S3A	Multiple Wilcoxon tests between control and doxycycline conditions, adjusting p-value using the two-stage step-up method of Benjamini, Kreiger and Yukutieli.		EGFP: q-value = 0.820625
Figure S3B	Multiple Wilcoxon tests between control and doxycycline conditions, adjusting p-value using the two-stage step-up method of Benjamini, Kreiger and Yukutieli.		rtTA: q-value = 0.75750<br>EGFP OE: q-value = 0.50500<br>FOXO1 OE: q-value = 0.883750<br>ASCL1 OE: q-value = 0.841667
Figure S3C	Multiple Wilcoxon tests between control and doxycycline conditions, adjusting p-value using the two-stage step-up method of Benjamini, Kreiger and Yukutieli.		rtTA: q-value = 0.820625<br>EGFP OE: q-value = 0. 820625<br>FOXO1 OE: q-value = 0. 820625<br>ASCL1 OE: q-value = 0. 820625
Figure S3D	Multiple Wilcoxon tests between control and		rtTA: q-value = 0.252500<br>EGFP OE: q-value = 0. 252500

Development • Supplementary information

	doxycycline conditions, adjusting p-value using the two-stage step-up method of Benjamini, Kreiger and Yukutieli.		FOXO1 OE: q-value = 0. 252500<br>ASCL1 OE: q-value = 0. 631250
Figure S3E	Multiple Wilcoxon tests between control and doxycycline conditions, adjusting p-value using the two-stage step-up method of Benjamini, Kreiger and Yukutieli.		EGFP control vs EGFP DOX: q-value = 0.063125
Figure S3F	Multiple Wilcoxon tests between control and doxycycline conditions, adjusting p-value using the two-stage step-up method of Benjamini, Kreiger and Yukutieli.		rtTA: q-value = 0.883750<br>EGFP OE: q-value = 0. 883750<br>FOXO1 OE: q-value = 0.883750<br>ASCL1 OE: q-value = 0. 883750
Figure S3G	Multiple Wilcoxon tests between control and doxycycline conditions, adjusting p-value using the two-stage step-up method of Benjamini, Kreiger and Yukutieli.		rtTA: q-value = 0.25250<br>EGFP OE: q-value = 0. 441875<br>FOXO1 OE: q-value = 0. 441875<br>ASCL1 OE: q-value = 0. 441875
Figure S3H	Multiple Wilcoxon tests between control and doxycycline conditions, adjusting p-value using the two-stage step-up method of		rtTA: q-value = 0.589157<br>EGFP OE: q-value = 0. 589157<br>FOXO1 OE: q-value = 0. 589157<br>ASCL1 OE: q-value > 0. 999999

Development • Supplementary information

	Benjamini, Kreiger and Yukutieli.		
Figure 4K	Row matched one-way ANOVA	$F_{(1.245, 3.734)}$ = 46.00 p-value = 0.0029	Tukey's multiple comparisons comparing the mean of every column.<br>EGFP OE vs FOXO1 OE: adjusted p-value = 0.0845<br>EGFP OE vs ASCL1 OE: adjusted p-value = 0.0177<br>FOXO1 OE vs ASCL1 OE: adjusted p-value = 0.0090
Figure 4M	Ordinary one-way ANOVA	$F_{(2, 9)}$ = 5.475 p-value = 0.0278	Tukey's multiple comparisons comparing the mean of every column.<br>EGFP OE vs FOXO1 OE: adjusted p-value = 0.0225<br>EGFP OE vs ASCL1 OE: adjusted p-value = 0.3198<br>FOXO1 OE vs ASCL1 OE: adjusted p-value = 0.2339
Figure 4P	Paired t-test	p-value = 0.0063	
Figure S5C	Paired t-test	p-value = 0.0085	
Figure S5G	Unpaired t-test	p-value = 0.0001	
Figure S5H	Unpaired t-test	p-value = 0.0064	
Figure S5I	Unpaired t-test	p-value = 0.0020	
Figure 5L	Ratio paired t-test	p-value = 0.0016	
Figure 5M	Ratio paired t-test	p-value = 0.1677	
Figure 5N	Ratio paired t-test	p-value = 0.1566	
Figure 5Q	Ratio paired t-test	p-value = 0.0013	
Figure S7D	Ratio paired t-test	p-value = 0.1208	
Figure 6K	Row matched one-way ANOVA	$F_{(2.919, 23.35)}$ = 6.614 p-value = 0.023	Tukey's multiple comparisons<br>Comparisons — Adjusted p value<br>0 vs 2 — 0.7433

Development • Supplementary information

			0 vs 4	0.1103
			0 vs 6	0.4186
			0 vs 10	0.3949
			2 vs 4	0.0135
			2 vs 6	0.0176
			2 vs 10	0.0836
			4 vs 6	0.9367
			4 vs 10	0.7635
			6 vs 10	0.9997
Figure 6L	Row matched one-way ANOVA	$F_{(2.552, 20.41)}$ = 6.812 p-value = 0.0033	Tukey's multiple comparisons	
			Comparisons	Adjusted p value
			0 vs 2	0.0077
			0 vs 4	0.9996
			0 vs 6	>0.9999
			0 vs 10	0.2949
			2 vs 4	0.1994
			2 vs 6	0.1015
			2 vs 10	0.0092
			4 vs 6	0.9976
			4 vs 10	0.5795
			6 vs 10	0.2865
Figure 6M	Row matched one-way ANOVA	$F_{(2.309, 18.48)}$ = 5.104 p-value = 0.0141	Tukey's multiple comparisons	
			Comparisons	Adjusted p value
			0 vs 2	0.0661
			0 vs 4	0.9600
			0 vs 6	0.8618
			0 vs 10	0.9972
			2 vs 4	0.0416
			2 vs 6	0.2422
			2 vs 10	0.1702
			4 vs 6	0.9753
			4 vs 10	0.9968
			6 vs 10	0.8965
Figure 6N	Ordinary two-way ANOVA	Species: $F_{(1, 80)}$ = 14.86, P=0.0002 Timepoint: $F_{(4, 80)}$ = 1.8, P=0.1349 Interaction: $F_{(4, 80)}$ = 1.9,	Šídák's multiple comparisions	
			Comparisons	Adjusted p value
			0	0.9014
			2	0.0005
			4	0.9830
			6	0.3795
			10	0.6350

Development • Supplementary information

		P=0.1127	
Figure 7D	Ratio paired t-test	p-value = 0.0233	
Figure 7E	Ratio paired t-test	p-value = 0.2703	
Figure 7F	Ratio paired t-test	p-value = 0.0749	
Figure 7I	Ratio paired t-test	p-value = 0.0097	

Table S10. Transfer vectors used in this study

Transfer vector	Reference	Catalogue number
Tet-O-FUW-Ascl1	Liu *et al (Liu et al., 2018)*	Addgene cat# 27150
Tet-O-FUW-Foxo1	Liu *et al (Liu et al., 2018)*	Addgene cat# 118588
FUW-tetO-EGFP	Panciera *et al (Panciera et al., 2016)*	Addgene cat# 84041
Lenti-SFFV-mNG-DamAlone	Lim *et al (Lim et al., 2023)*	N/A
Lenti-SFFV-mNG-Dam-Ascl1	This paper	N/A
Lenti-SFFV-mNG-Foxo1-Dam	This paper	N/A
pLV-EF1a-IRES-Puro	Hayer *et al (Hayer et al., 2016)*	Addgene cat# 85132
Scramble shRNA	Sarbassov *et al (Sarbassov et al., 2005)*	Addgene cat# 1864
pLKO.1	Moffat *et al (Moffat et al., 2006)*	Addgene cat# 10878

Development • Supplementary information
